# Oxidation of dibenzothiophene (DBT) by *Serratia marcescens* UCP 1549 formed biphenyl as final product

**DOI:** 10.1186/1754-6834-5-33

**Published:** 2012-05-14

**Authors:** Hélvia W Casullo de Araújo, Marta Cristina de Freitas Siva, Clarissa I Matos Lins, Aline Elesbão do Nascimento, Carlos Alberto Alves da Silva, Galba M Campos-Takaki

**Affiliations:** 1 Departamento de Química, Universidade Estadual da Paraíba (UEPB), Campina Grande, PB, 58429-500, Brazil; 2 Núcleo de Pesquisas em Ciências Ambientais (NPCIAMB), Universidade Católica de Pernambuco (UNICAP), Recife, PE, 50.050-900, Brazil; 3 Doutorado em Engenharia Química, Faculdade de Engenharia Química, Universidade Estadual de Campinas, Campinas, SP, 13083-970, Brazil; 4 Departamento de Química, Universidade Católica de Pernambuco (UNICAP), Recife, PE, 50.050-900, Brazil

## Abstract

**Background:**

The desulphurization of dibenzothiophene (DBT), a recalcitrant thiophenic fossil fuel component by S*erratia marcescens* (UCP 1549) in order for reducing the Sulphur content was investigated. The Study was carried out establishing the growth profile using Luria Bertani medium to different concentrations of DBT during 120 hours at 28°C, and orbital Shaker at 150 rpm.

**Results:**

The results indicated that concentrations of DBT 0.5, 1.0 and 2.0 mM do not affected the growth of the bacterium. The DBT showed similar Minimum Inhibitory Concentration (MIC) and Minimum Bactericidal Concentration (MCB) (3.68 mM). The desulphurization of DBT by *S. marcescens* was used with 96 hours of growth on 2 mM of DBT, and was determined by gas chromatography (GC) and GC-mass spectrometry. In order to study the desulphurization process by *S. marcescens* was observed the presence of a sulfur-free product at 16 hours of cultivation.

**Conclusions:**

The data suggests the use of metabolic pathway “4S” by *S. marcescens* (UCP 1549) and formed biphenyl. The microbial desulphurization process by *Serratia* can be suggest significant reducing sulphur content in DBT, and showed promising potential for reduction of the sulfur content in diesel oil.

## Background

Dibenzothiophene (DBT) is a heterocyclic molecule containing sulfur present in petroleum and its degradation has been studied as the model organic sulfur compound in crude oil [1]. There are three pathways for the biodegradation of DBT: Kodama, the Van afferden and 4S routes [2-4]. The petrochemical sector has important and specific characteristics with respect to effluent, with pollutants from chemical composition is extremely variable, such as polycyclic aromatic hydrocarbons (PAH’s), organ sulfur compounds and heavy metals [5]. The dibenzothiophene (DBT) shows high toxicity and mutagencity may affect human health and ecosystems in general [6].

However, the presence of these elements is undesirable not only contributing to corrosion of equipment in the refinery, but also when ignited, they release sulfur dioxide (SO_2_) as a major air pollutant responsible for acid rain [7,8]. In big cities, for example, about 40% of air pollution resulting from burning gasoline and diesel oil for vehicles, responsible for the issue of carbon monoxide and carbon dioxide, nitrogen oxides, sulfur dioxide, hydrocarbons and lead derivatives [9,10]. The sulfur content in fuels is continuously being reduced by regulations, to the lowest levels ever. Europe and the United States, currently require a maximum of 50 ppm sulfur in gasoline and diesel and this level will be reduced to less than 10 ppm in 2010 [11].

To remove sulfur from fossil fuels, refiners have a technique hydrodesulfurizing (HDS), which is much cost, energy-intensive and do not effective in removing hydrocarbons, and especially sulfur compounds, such as dibenzothiophene. The bulk of inorganic sulphur and simple organic sulphur can be removed by HDS but this process is proving to be inadequate to produce low-sulphur fuels as it is unable to remove the complex polycyclic sulphur compounds [12].

Therefore, the microbial desulfurization or biodesulphurization (BDS) has attracted attention for being a more efficient alternative, low cost, which uses microorganisms to desulphurizer these compounds, promoting selective metabolism of sulfur (attacking C -S) without degrading the carbon skeleton (C - C), keeping the energy source of the molecule intact [13]. The dibenzothiophene is considered a model compound for studies of biological desulfurization of fossil fuels and for studies of persistent compounds such as S-heterocycles in the environment [14,15]. Several microorganisms have been studied for removal of sulfur biochemistry of DBT [16]. Prokaryotic organisms that desulphurizer organ sulfur compounds without metabolizing the carbon skeleton are uncommon and are usually used in the process of selective oxidation of sulfur [17,18].

Considering the relevance of the overall impact of pollution generated of organ sulfur compounds from diesel, we report in this investigation the kinetic model of the growth of the *S. marcescens* (UCP 1549) on DBT, determination of MIC and MBC*,* and the desulphurization of DBT using metabolic pathway.

## Results

### Effect of DBT on the growth of S*erratia marcescens*

**Figure 1:**
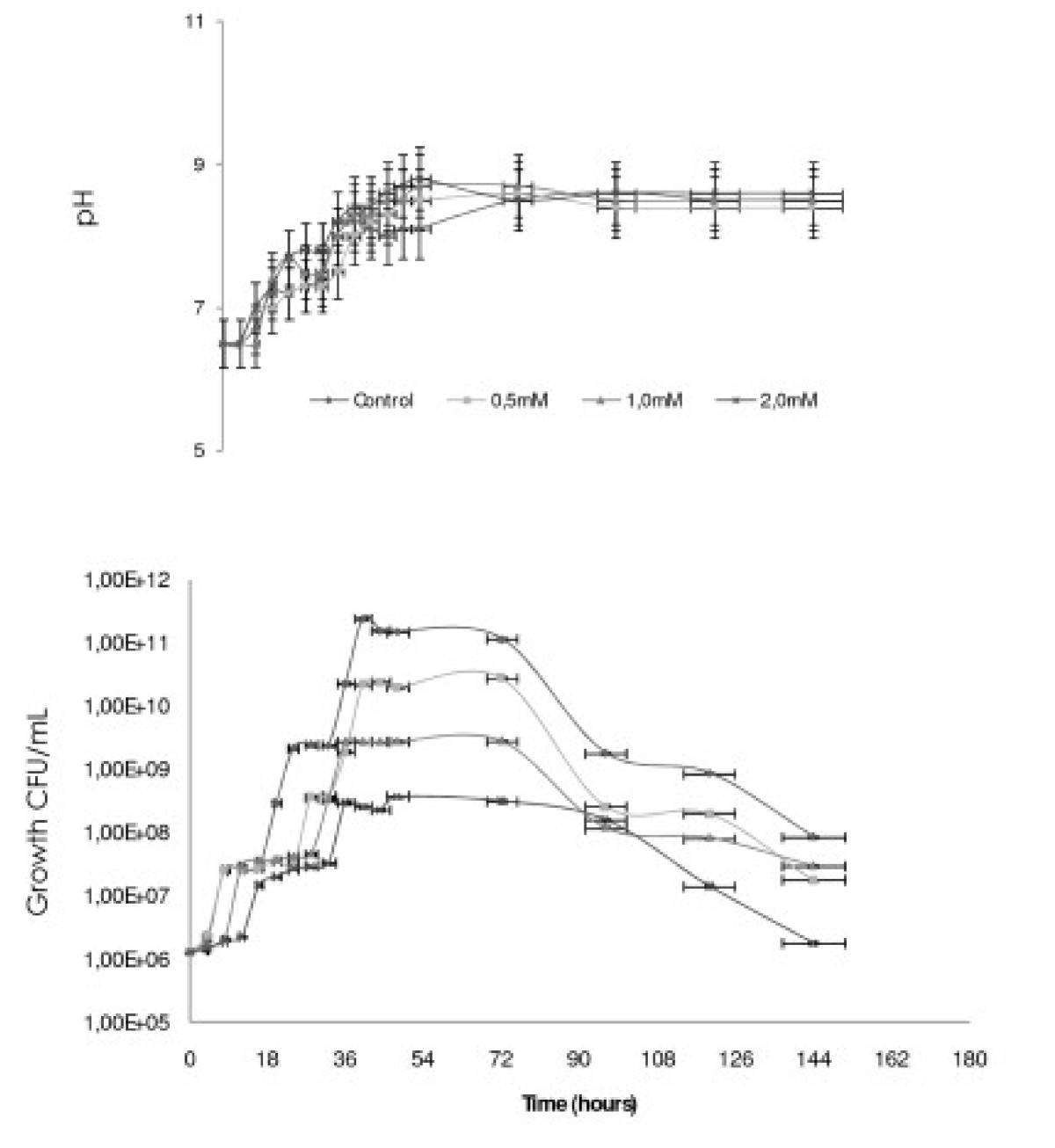
**Growth profile of the S** ***erratia marcescens *** **in Luria Bertani (LB) containing 0.0 0.5, 1.0 and 2.0 mM DBT, under orbital agitation 150 rpm, at 28°C during 144 hours.**

The growth profile by *S. marcescens* (UCP1549) Showed a Short “lag phase” of growth early in the first 4 hours, followed the exponential growth phase at 8 hours. The maximum growth was observed after 36 hours of incubation followed by a small stationary phase until 72 hours, followed by the decline phase. The maximum growth μMax was 0.324 h^-1^ with the generation time (T_G_) at 2.12 hours (Table 1). The pH of the growth variety to 6.5 until 8.5 (Figure 1).

**Table 1:** **Kinetic of growth of *Serratia marcescens *** **(UCP 1549) on the absence and different concentrations of dibenzothiophene (DBT), using Luria Bertani medium at 28°C, 150 rpm**

**Conditions**	**μ**_**esp**_**(h**^**-1**^**)**	**T**_**G**_**(h)**
*Control	0.324	2.12
**0.5 Mm**	0.150	4.60
**1.0 mM**	0.110	6.27
**2.0 mM**	0.050	13.80

*Without DBT.

Kinetics of the growth of *S. marcescens* (UCP 1549) in DBT concentrations (0.5, 1.0 and 2.0 mM) (Figure 1), there was a Slight inhibition in the period 0–8 hours of growth for the concentration of 2.0 mM. However, the concentrations of DBT (0.5 and 1.0 mM) showed inhibitory effect in the early of exponential phase. In addition, the major concentrations of 1.0 and 2.0 mM were observed action in the top of exponential growth phase at 12 hours of cultivation. The specific growth rates were 0.15 h^-1^, 0.11 and 0.05 h^-1^ with the generation time (T_G_) of 4.6, 6.27 and 13.8 hours, based in the response of the concentrations 0.5, 1.0 and 2.0 mM of DBT, respectively (Table 1).

### Susceptibility and ultra structural analyses of *S. marcescens* to DBT

The DBT Showed low inhibitor power for the growth of *S. marcescens*. The results indicated a 3.68 mM concentration as MIC. And, similar results were observed to the Minimum Bactericidal Concentration (MCB).

**Figure 2:**
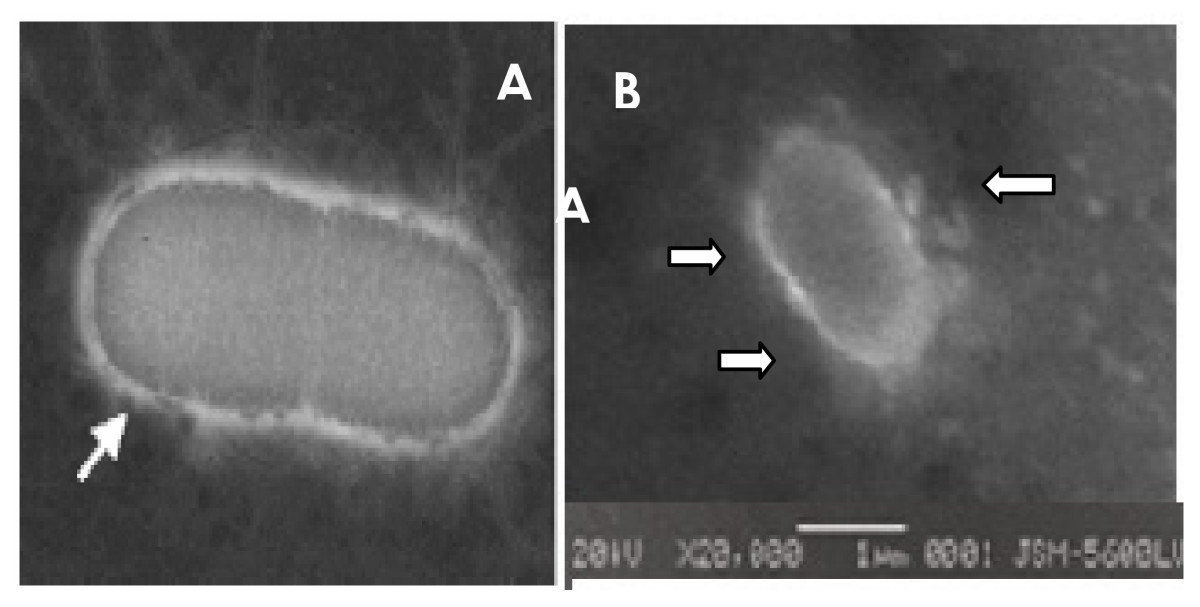
***Serratia marcescens *** **strain by transmission electron microscopy [**[19]**] (A); Electron micrographs of *Serratia marcescens *** **(UCP1549) and shown high electron density points in the cell surface (x 20.000)-(B).**

The ultra structural analysis of *S. marcescens* UCP 1549 using transmission electron microscopy Showed the existence an outer layer similar to an electron-lucent ring around the cell (Figure 2) suggesting the presence of capsular polysaccharides or lipopolysaccharides.

### Removal and Desulfurization of DBT by *Serratia marcescens*

The results obtained with DBT removal by *S. marcescens* (UCP 1549) indicating that the concentration decreases at 24 hours of incubation. In experiment was observed a removal of 100% after 96 hours of incubation with 0.5 and 2.0 mM DBT concentrations, except to 1 mM. This concentration of 1.0 mM DBT was removed later at 144 hours of cultivation (Table 2).

**Table 2:** **Kinetic of removal dibenzothiophene (DBT) by S** ***erratia marcescens *** **during the growth in Luria Bertani medium**

**Conditions**	**Kinetic of DBT removal (Velocity) (%/Hours)**			
	**μ**_**esp**_**(h**^**-1**^**) 96 120 144**			
*Control	–	–	–	–
**0.5 mM**	0.038	50.00	100	100
**1.0 mM**	0.022	84.30	97.80	100
**2.0 mM**	0.038	98.00	99.05	100

*Without DBT-(0% of DBT removal).

**Figure 3:**
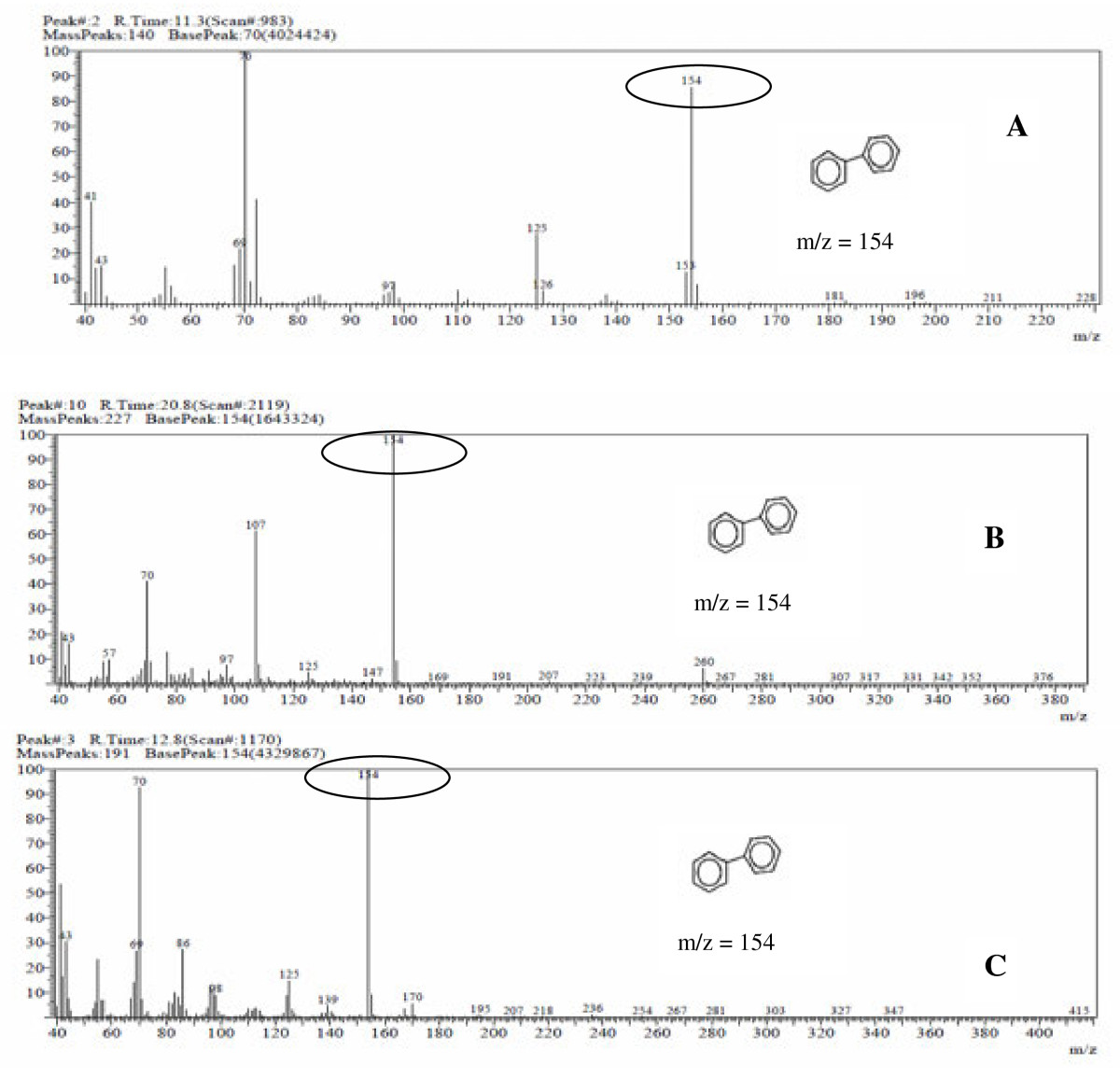
**Mass spectrogram of the metabolite formed in the degradation of 2 mM of dibenzothiophene DBT by S*erratia marcescens *UCP (1549) at 20°C.** Transformation of biphenyl (m/z = 154). (**A**) - conversion in 90% of biphenyl to 11.3 min for 16 hours of fermentation, (**B**) - 100% of biphenyl in 24 hours of fermentation, (**C**) - 100% of biphenyl in 144 hours of fermentation.

To study the DBT utilizing ability of the bacteria in liquid medium cultures, the amount of DBT was measured in the cultures. *S. marcescens* grown at 2.0 mM of DBT did not showed the presence of DBT, confirming that the DBT was metabolized before the first 24 hours of growth (Figure 3).

The metabolites formed by the degradation of dibenzothiophene when analyzed of GC-MS showed that the sulfur atom was removed from the DBT. In addition, the peak formed correspond to a biphenyl compound, which corresponds to loss of sulfur and formation of groups of phenol (Figure 3), suggesting perhaps the formation of sulfates.

**Figure 4:**
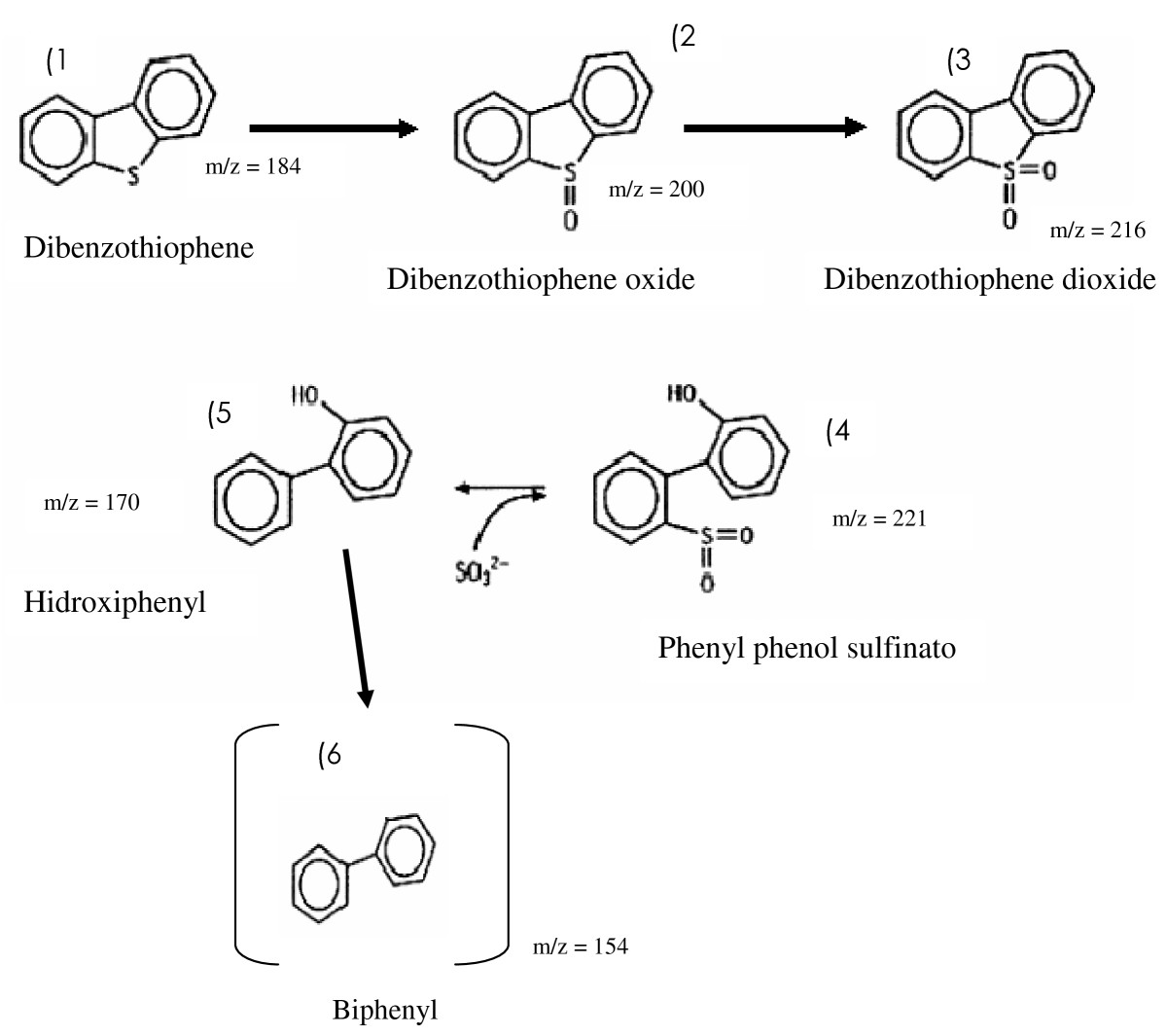
**Proposed (so called 4S) pathway for DBT desulfurization by *Serratia marcescens *(UCP 1549).** The DBT desulfurization pathway results: (1) dibenzothiophene, (2) dibenzothiophene oxide, (3) dibenzothiophene dioxide, (4) sulfinato phenyl phenol, (5) hydroxyphenyl (6) biphenyl. The structure on parentheses is the metabolite detected, other structures are suggested, not detected.

The formation of a compound free of sulfur (biphenyl), the retention time at 11.3 min, was detected with 85% at 16 hours of cultivation (Figure 4 and 4A). The retention at 12.8 min was detected with 100% (Figure 3), the desulfurization and formation of biphenyl was determined by the continuity of culture from 24 to 120 hours at 28°C in the concentration of 2 mM DBT (Figure 3).

These results suggest that bacterial metabolism of dibenzothiophene, DBT by *S. marcescens* when analyzed by GC-MS, obtaining as the final metabolite biphenyl (Figure 3). The results suggest that the metabolic pathway used by *S. marcescens* in the biodesulfurization process with 2 mM of DBT, indicated the specific via of the 4S, transformation of DBT in dibenzothiophene oxide, followed dibenzothiophene dioxide, sulfinato phenyl phenol, 2-hidroxybifenil, and final product biphenyl as the proposedScheme (Figure 4).

## Discussion

The cells by *Serratia marcescens* remained viable in the presence of DBT in the concentrations of 0.5, 1.0 and 2.0 mM, and showed similar behavior when compared with the growth control (without DBT). This fact is probably due to the compound DBT is considering essential for amino acids the formation, such as cysteine, cystine, methionine, some vitamins and other compounds important to the survival of microorganisms [13,20,21].

Additionally, the literature cites the importance of the structure in the abilities of cell motility as well as the identification of serotypes [22,23]. The results presented in this study with the use of scanning electron microscopy corroborate the data presented in the topological analysis showing the existence of an electron-lucent layer on the surface by *S. marcescens*. However, the cultivation in the presence of DBT did not show variations using the routine technique in cellular topology.

Organic compounds as DBT containing sulfur are a small but important fraction of some fuel and due to their difficult biodegradability are considered recalcitrant compounds. The presence of sulfur is undesirable because it is contributing to the corrosion of equipment in the refinery. However, the environmental pollution is caused by release of the emission of sulfur dioxides into the atmosphere on combustion of sulphur-containing fossil fuels. These environmental pollution lead to a health problems as: potential of acid rain and carcinogenicity to humans [6,24,25].

The sulfur content in fuels is continuously being reduced by regulations, however increasingly low levels in several countries, currently the U.S.A. and Europe require a maximum of 50 ppm with a pretense reduction to less than 10 ppm of the end of 2010 [11].

The refineries and chemical process called hydrodesulphurization (HDS) used for removal of inorganic sulfur. These treatments are costly, involving chemical catalytic treatment under extreme conditions (200–425°C), and higher pressures 150 to 205 psi [26,27]. The inorganic sulfur and organic sulfur can be removed simply HDS, but this process is unsuitable for production of fuels with low sulfur content. Since this process is unable to remove sulfur compounds from complex hydrocarbons containing sulfur, present in oil and coal. Thus, thiophene compounds represent a large amount of sulfur after treatment of HDS for fuel [4,24].

One strategy for reducing the sulfur content of these substrates is to expose these substrates to microorganisms that can specifically break carbon-sulfur chain, releasing sulfur to water-soluble portion, in inorganic form. This process of microbial or biodesulphurization (BDS) is an attractive alternative compared to the HDS as due to the mild operating conditions (low pressure and temperature) and reaction specificity afforded by the biocatalyst becomes an effective technique and of low cost [21,28].

Thus, the new information of pathway used by *S. marcescens* UCP1549 transformation of 2 mM of DBT, possibly via the use of BDS 4 S, is based on product formed identified by their mass spectra detected by GC-MS analysis, which fragmented ion (m/z = 154) corresponding to biphenyl, according to spectral Database for Organic Compounds SDBS library.

BDS based on the 4S pathway, is a specific desulfurization pathway in the conversion of DBT into 2-HBP. In this way the carbon skeleton of DBT is released intact, and so the calorific value of fuel is not lost. This route is high energy because the carbon skeleton is not mineralized. The pathway 4S is a complex system of enzymes and cofactors and their requirements prohibit the use of systems for purification of enzymes and not the whole cell that transforms into a practical process of BDS [29]. They also have free cell extracts at a lower desulfurization activity [30,31].

The implementation of the BDS process via 4S consists of several stages, including (i) growth of the selected strain in a suitable environment in order to obtain cells that have the highest desulfurization activity possible and (ii) removal of active cells by cells at rest (stationary). The formulation of the growth medium is important for the production of a high density of resting cells, which expressed the highest level of desulfurization activity [32].

Researchers showed that the presence of sulfates on the medium where cells grown in DBT does not inhibit the activity of desulfurization towards 4S, thus allowing the proliferation of cells (stationary phase), which also does not inhibit this process (stationary phase), which also does not inhibit this process [28,32,33]. The specific route of the sulfur can be accomplished by aerobic and anaerobic bacteria. Sulfate-reducing bacteria have been reported to desulphurizer model compounds (DBT) and fossil fuels [34,35]. However, no significant reduction in the sulfur content of DBT. Currently, there is evidence for a potential anaerobic desulfurization commercially significant, thus, the aerobic BDS has been the focus of most researchers [5,28]. The use of this pathway has been proposed for the desulfurization of oil production fields to refineries and also [15,28,32].

The DBT 5,5—dioxide is involved in the process of biodesulfurization by 4S [36,37]. Pathways of degradation of DBT by bacteria, has been widely studied, as to: *Arthrobacter*[38], *Brevibacterium*[39], *Mycobacterium*[14,15,40]. *Brevibacterium* sp. DBT mineralized completely, using it as a source of carbon, sulfur and energy, forming as final compounds: benzoic acid, sulfite and water via deoxygenating known as angular [39]. Under aerobic conditions, 3-hydroxy-2-formyl benzothiophene was formed from the degradation of DBT by the bacterium *Pseudomonas* sp. [21], 1,2-dihydroxy-1,2-dihidrodibenzothiophene and dibenzothiophene 5-oxide were generated by *Beijeninckia* sp [41]. Under anaerobic conditions, the biphenyl compound was formed by *Desulfovibrio desulfuricans* M6 (2). *Corynebacterium* sp. SY1 and *Rhodococcus rhodochrous* IGTS8 were only able to remove sulfur of DBT converting to compound hidroxybifenil 2-(2-HBP) [42]. These microorganisms have the ability to selectively remove organic sulfur without degrading the carbon atoms [33,36,43]. The removal of sulfur by *Paenibacillus* sp A11-2 as a carbon source using methyl, ethyl, dimethyl, trimethyl and propyl DBT was obtained as final product hydroxylated biphenyls [28,40,44,45]. The hydrocarbon was desulphurizer DBT by growing cells by *Pseudomonas* sp and others microorganisms [42,46,47], as well as by growth and stationary phase cells at 30°C, *Gordona s*p. [12,48]. The process of HDS of DBT combined lithium (Li) and sodium (Na) in the system and obtained a yield of biphenyl 88% [6]. The consumption of chemicals would result in higher costs [27].

## Conclusions

In this study, BDS by *S. marcescens* (UCP 1549), the 2 mM desulphurizer and DBT was converted to biphenyl, a sulfur-free product in 16 hours of cultivation with 100% staying the product formed in the other culture. The biodesulphurization of DBT converted to 2-HBP may increase the possibility of environmental pollution. In contrast, the conversion of biphenyl to 2-HBP can partially eliminate the inhibitory effect of products and pollution from the combustion of diesel oil.

The ability of DBT biodesulphurization may be formed instable metabolites or by the existence of additional pathways(s) on the degradation of DBT. The removal of sulfur compounds by BDS occurs in most aromatic in the relatively low molecular weight. In conclusion, we demonstrated the removal of sulfur from DBT by *S. marcescens* (UCP 1549) and led to a substantial decrease in the total sulphur content and produce biphenyl compound. As more efficient desulphurization was observed, and the study indicates that treatment of diesel oil, considering the possibility of the production of ultra-low sulphur fuel in accordance with the regulations. In addition, we described the specific via of sulfur- degradation with selective cleavage of C-S union mediated by *S. marcescens*. Although it appears that the *S. marcescens* is capable of desulphurizer the DBT, and the desulfurization with formation of biphenyl was determined by the continuity of culture from 24 to 120 hours at 28°C in the concentration of 2 mM DBT. However, the formation of that compound still required a lot of research to discover the real mechanism of the metabolic pathway of desulphurization of DBT.

## Methods

### Microorganism

This experiment used a pure culture by *Serratia marcescens* (UPC1549) isolated from the semi-arid soil of northeastern region of Brazil (Pernambuco - Brazil) banana culture, deposited in the Culture Collection of the Nucleus of Research in Environmental Sciences, Catholic University of Pernambuco, registered in the World Federation Culture collection (WFCC). The bacterium was maintained in solid nutrient agar medium at 4°C.

### Culture medium

The bacterium was grown on Luria Bertani medium-LB [tryptone (10.0 g), yeast extract (5.0 g), NaCl (10.0 g) and glucose (5.0 g) per liter], as a supplement.

### Dibenzothiophene (DBT)

The DBT was purchased from Aldrich, cat: D3, 220–2 and prepared a stock solution in NN-dimethylformamide at a concentration of 1 M (w/v), the solution was sterilized in Millipore ® filter [49].

### Culture conditions

The growth of *S. marcescens* was performed in Erlenmeyer flasks of 250 ml of capacity containing 100 ml of LB in DBT concentrations (0.0, 0.5, 1.0 and 2.0 mM), and 1 mL of pre-inoculum of 10^6^/mL was transferred. The flasks were maintained in orbital shaker at 150 rpm, temperature of 28°C. All experiments were performed in triplicate. Each 4 hours, aliquots were removed to determine the number of viable cells (UFC/mL) until 48 hours, followed to collect each 24 hours until 144 hours of cultivation. Bacterial growth was evaluated by the units colonies formed (UCF/mL) using the technique “Pour plate”. The specific velocity of growth (μh^-1^) and the generation time (T_G_) were determined by Pirt [50].

For the specific growth rate equation used was:$\mu_{\text{esp}}=\left(\text{LnX}–{\text{LnX}}_{0}\right)/\left(T-{\text{T}}_{0}\right)$ Eq (1)

Where: X = value of the exponential growth phase X_0_ = early exponential phase T = time the exponential growth phaseT_0_ = initial time of the exponential phase.

The generation time (T_G_) was determined by the formula: $T_{G}=\text{Ln}2/\mu_{\text{esp}}$ Eq (2)

### Determination of Minimum Inhibitory Concentration (MIC) and Minimum Bactericidal Concentration (MBC)

The minimum inhibitory concentration (MIC) of DBT was carried out using quantitative method of dilution in liquid medium, considered the reference to determine the sensitivity by *S. marcescens* against the different concentrations of DBT (0.0, 0.5, 1.0 and 2.0 mM). Progressive dilutions of DBT (0.5–0.01 mL) were added to LB broth medium. The suspension of the bacterium with 16 hours of growth in LB broth, corresponding to 10^6^ UFC/ml was inoculated in test tubes DBT concentrations and the control (0) of DBT. The tubes were incubated at 28°C for 18 hours. After this period all tubes were determined the optical density at 600 nm comparing with control (0 of DBT). The MIC of DBT to *S. marcescens*(UCP 1549) was calculated based in the minus concentration of DBT caused total inhibition of the growth bacteria. The minimum bactericidal of DBT concentration was determined transferring 1 mL of the tubes test content to AN, and counted the viable colonies formed in comparison with the control (without DBT). The results of MBC were determined in relation to minimum concentration of DBT do not observed growth [51].

### Electron microscopy scanning

*S. marcescens* (UCP 1549) grown in Luria Bertani medium (control) or treated with dibenzothiophene (DBT) 2 mM were collected each 24 hours for a period of 120 hours.

The cells were washed in PBS (buffered saline) pH 7.2 for 10 minutes, and then fixed with 2.5% glutaraldehyde in phosphate buffer, 0.1 M, pH 7.4, for 1 hour at room temperature. The cells were again washed with PBS, twice for 10 minutes, and after fixing with osmium tetroxide of 1% in PBS for 1 hour at room temperature, in dark conditions. Then the cells were again washed with 0.1 M phosphate buffer, were then subjected to the dehydration process. For the dehydration of the samples was used ethyl alcohol in proportions of 50%, 70%, 90% (5 minutes for each change) until the ratio of 100% (three times, 10 minutes each change). After this step, the cells were subjected to critical point for the total elimination of the liquid phase, followed by mounting on aluminum poles and subsequent metallization. Thus prepared, the samples were analyzed and photographed in an electron microscope scanning (JEOL 5600 LV LSM) operating at 20 Kv [52]. Increase of 20.000 x.

### Analysis Gas Chromatography—Mass Spectrometry (GC-MS)

After fermentation, the samples were centrifuged at 5.000 g for 15 minutes at 5°C to separate the biomass fluid metabolism. From the metabolic liquid extract, was performed for chromatography to determine the compounds formed from the metabolic pathway used by *S. marcescens* to degrade DBT at 2 mM concentration for a period of 120 hours, was extracted with equal volume of ethyl acetate (60 mL/60 mL), aliquots were taken every 24 hours. The organic layer was removed and the aqueous layer was acidified to pH 2.0 with 5 N HCl solution, extracted with an equal volume of ethyl acetate [53].

The extracts were vacuum rotator evaporator and re-suspended in 2 mL of ethyl acetate and then analyzed by gas chromatography coupled to mass spectrometry (GC-MS Shimadzu QP5050A). The chromatographic conditions were: injector temperature exceeding 250°C initial temperature of the furnace equal to 60°C, rate of variation of oven temperature of 10°C/min final temperature of the furnace equal to 280°C, helium gas was used as phase mobile (1 mL/min); Splitter ratio equal to 1:60; DB-5 column (30 m × 0.257 mm × 0.25 mm). The mass spectrum was obtained for a region of m/z 40 to 650.

### Determination of removal of DBT

The level of removal of DBT was determined on UV-visible using a calibration curve with 1–10 mM DBT a solution, at a wavelength of 250 nm. Aliquots each 24 hours were collected of metabolic liquid obtained from the growth of *S. marcescens* on different concentrations of DBT (0, 0.5, 1.0 and 2.0 mM) at temperature of 28°C and 150 rpm for 144 hours [54].

## Abbreviations

BDS: Biodesulphurization; C: Carbon; DBT: Dibenzothiophene; GC: Gas chromatography; GC – MS: Gas chromatography–mass spectrometry; h: hours; HBP: Hidroxybifenil; HCl: Hydrochloric acid; HDS: Hydrodesulfurizing; LB: Luria Bertani; Li: Lithium; LN: Logarithm neperian; m: meter; M: Molar; MCB: Minimum Bactericidal Concentration; MIC: Minimum Inhibitory Concentration; Min: Minutes; mM: Millimolar; Na: Sodium; Na Cl: Sodium chloride; Nm: Nanometer; Ppm: Parts per million; Rpm: Rotations per minutes; S: Sulfur; SDBS: Spectral Database for Organic Compounds; SO_2_: Sulfur dioxide; T_G_: Generation time; U.S.A: UnitedStates of America; UCP: Catholic University of Pernambuco; UV - visible: Ultraviolet visible; WFCC: World Federation Culture collection.

## Competing interests

The authors declare that they have no competing interests.

## Authors’ contributions

HWCA obtained the kinetic of growth, Minimum Inhibitory Concentration (MIC) and Minimum Bactericidal Concentration (MIB), and the evaluation of DBT degradation by gas chromatography (GC) and GC-mass spectrometry, and the progress of the experimentation. MCFS and CIML contributed with DBT removal using spectrophotometer method. AEN was done the electron microscopy method for identification. GMCT helped draft the manuscript, advised on the design, and contributed to the original conception of this study. All authors critically revised the draft and approved the final manuscript.
